# Comparison of Four Methods of RNA Extraction and cDNA Synthesis from The Venom of Peruvian Snakes of the Genus *Bothrops* of Clinical Importance

**DOI:** 10.3390/ijms241311161

**Published:** 2023-07-06

**Authors:** Daniel Torrejón, Javier Cárdenas, Diana Juárez, Jordano Espinoza, Alex Proleón, Andrés Agurto-Arteaga, Fanny Lazo, Mariana Leguía, Félix A. Urra, Eladio F. Sánchez, Carlos Chávez-Olortegui, Dan E. Vivas-Ruiz, Armando Yarlequé

**Affiliations:** 1 Laboratorio de Biología Molecular, Facultad de Ciencias Biológicas, Universidad Nacional Mayor de San Marcos, Av. Venezuela Cdra 34 S/N, Ciudad Universitaria, Lima Cercado, Lima 15081, Peru; 2 Laboratorio de Bioquímica, Facultad de Ciencias de la Salud, Universidad Nacional del del Callao, Av. Juan Pablo ΙΙ 306, Bellavista 07011, Peru; 3 Laboratorio de Genómica, Pontificia Universidad Católica del Perú, Av. Universitaria 1801, Campus Principal, San Miguel 15088, Peru; 4 Laboratorio de Plasticidad Metabólica y Bioenergética, Programa de Farmacología Clínica y Molecular, Instituto de Ciencias Biomédicas, Facultad de Medicina, Universidad de Chile, Santiago 8380453, Chile; 5 Network for Snake Venom Research and Drug Discovery, Av. Independencia 1027, Santiago 7810000, Chile; 6 Research and Development Center, Ezequiel Dias Foundation, Belo Horizonte 30510-010, Minas Gerais, Brazil; 7 Departamento de Bioquímica-Inmunología, Instituto de Ciências Biológicas, Universidade Federal de Minas Gerais, Belo Horizonte 31270-901, Minas Gerais, Brazil

**Keywords:** *Bothrops*, venom, RNA purification, cDNA synthesis, venom gland, transcripts

## Abstract

RNA purification and cDNA synthesis represents the starting point for molecular analyses of snake venom proteins-enzymes. Usually, the sacrifice of snakes is necessary for venom gland extraction to identify protein-coding transcripts; however, the venom can be used as a source of transcripts. Although there are methods for obtaining RNA from venom, no comparative analysis has been conducted in the *Bothrops* genus. In the present study, we compared four commercial methods for RNA purification and cDNA synthesis from venom (liquid, lyophilized, or long-term storage) of four clinically relevant species of Peruvian *Bothrops*. Our results show that the TRIzol method presents the highest yield of RNA purified from venom (59 ± 11 ng/100 µL or 10 mg). The SuperScript First-Strand Synthesis System kit produced high amounts of cDNA (3.2 ± 1.2 ng cDNA/ng RNA), and the highest value was from combination with the Dynabeads mRNA DIRECT kit (4.8 ± 2.0 ng cDNA/ng RNA). The utility of cDNA was demonstrated with the amplification of six relevant toxins: thrombin-like enzymes, P-I and P-III metalloproteinases, acid and basic phospholipases A2, and disintegrins. To our knowledge, this is the first comparative study of RNA purification and cDNA synthesis methodologies from *Bothrops* genus venom.

## 1. Introduction

Pit vipers from *Bothrops* snakes are the most clinically relevant group in Latin America [1,2]. *Bothrops* venoms are composed of highly specialized proteins responsible for triggering coagulopathies, hemorrhage, myo/dermonecrosis, thromboinflammation, and pathologies [3,4]. These effects are caused by the synergic actions of protein families such as metalloproteinases (SVMP), serine proteinases (SVSP), L-amino acid oxidases (L-AAO), classical phospholipases A2 (PLA2), PLA2 homologs (PLA2-like), and disintegrins [5,6]. Therefore, the knowledge and understanding of the envenomation process depend on the functional and structural characterization of each member of these protein families. The routine methodology for the characterization of the proteins present in the venom is the isolation by different chromatographic methods followed by biochemical, biological, and immunological characterization [6,7]. On the other hand, molecular biology techniques have expanded the structural characterization of venomous snake proteins, discovered novel genes encoding proteins, enzymes, or toxins, identified mutations of interest, and allowed the production of recombinant versions of those with biomedical or biotechnological potential. For this purpose, molecular biology requires messenger RNA (mRNA) or, consequently, complementary DNA (cDNA) [8] for inferring valuable structural and functional information currently available in GenBank, the Protein Data Bank, or the Snake Venom Database.

Snake venom glands are an important source for molecular venom characterization, as they contain the venom-producing cells and associated structures; it is also a rich resource for the identification of bioactive molecules for drug development. Many comprehensive multi-omics studies have used the venom gland [8,9]. However, the use of the glands encompasses two important aspects: the sacrifice of the specimens and the stability of the tissue; therefore, the studies must be carried out within the ethical aspects and, with this, ensure an adequate endowment of samples. Likewise, there are limitations to those endemic species without wide distribution, species that are not adapted to living in captivity, and species that are in a state of vulnerability or endangered (CITES species) [10]. For this reason, for more than 30 years, there was a need to develop methodologies that allow the culture of the cells of the venom glands [11], and that currently has led to the generation of organoids from the venom glands [12]. Unfortunately, such technologies are still scanty in developing countries where, coincidentally, snakebite cases are recurrent. Given this fact, the use of venom (fresh or lyophilized) as a source to obtain RNA has been an alternative for the molecular characterization of at least the main proteins involved in the envenoming and is a less destructive method because the sacrifice of animals is avoided [13].

A range of adapted RNA isolation methods from venom have been proposed according to the logistics available in each laboratory, such as guanidinium thiocyanate (GITC)-based organic extraction [14,15,16,17], and silica-membrane column [18,19], glass-fiber filter [20], and magnetic particle [21,22,23] technologies. These studies showed full-length venom protein cDNA sequences from venom-derived mRNA for rear-fanged snakes, elapid species (*Pseudechis porphyriacus*, *Aspidelaps lubricus*, and *Naja kaouthia*), and front-fanged vipers (mainly *Crotalus*, *Sistrurus*, and *Bitis* species) [13]. Recently a protocol to obtain targeted transcript sequence data from *Crotalus durissus cumanensis* has been performed [21]. Its strategy allows the use of lyophilized venom to obtain complete toxin sequences [21]. Nevertheless, there are few studies about venom-derived RNA quality isolated and its feasibility for molecular detection of snake venom toxins [10,13,22] that allows knowing, quantitatively, what is the yield of obtaining RNA from venom (whether fresh or lyophilized) also considering the combination of RNA extraction and cDNA synthesis methods.

Considering all available data as reference, our laboratory has been successfully characterizing several proteins from Peruvian *B. pictus*, *B. barnetti*, and *B. atrox* venoms at the molecular level, using venom-derived RNA [14,24,25,26,27]. Now, we believe that, in addition to molecular identification, venom-derived RNA has a potential for RNA-based technologies such as cDNA library construction, transcriptomics, gene expression analysis, and recombinant protein synthesis. However, developing an optimized protocol considering the routinary kits available in the region is necessary. For this reason, the aim of this work was to compare the efficiency and performance in obtaining venom-derived RNA, the synthesis of cDNA, and the usefulness of the RNA obtained to protein-coding regions amplification of four RNA purification and four cDNA synthesis kits from lyophilized or liquid venoms of four clinically relevant Peruvian species of *Bothrops* genus. This is the first work carried out on *Bothrops* venom, the genus of greatest medical importance in South America. Additionally, cDNA synthesis from some *Bothrops atrox* lyophilized venoms up to 30 years old was successful for transcript amplification.

## 2. Results

### 2.1. RNA Purification

**Figure 1:**
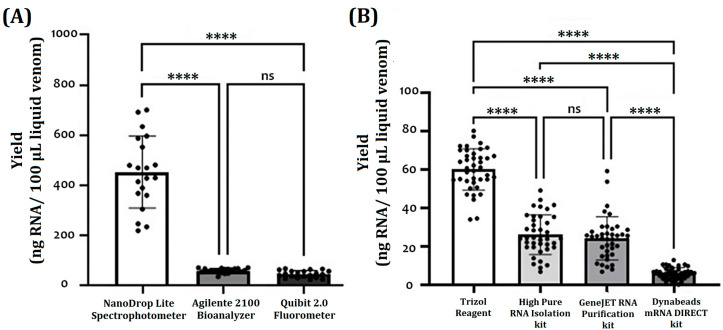
(**A**) Comparison of methods used for determination of RNA yield: NanoDrop Lite spectrophotometer, Qubit 2.0 fluorometer, and Agilent 2100 Bioanalyzer. Non-parametric Kruskal–Wallis test and multiple comparisons with Dunn’s test were used to assess the differences between the RNA quantification methods. (**B**) Comparison of methods used for RNA isolation: TRIzol Reagent, High Pure RNA Isolation kit, GeneJET RNA Purification kit, and Dynabeads mRNA DIRECT kit protocols. The RNA/cDNA concentration met the assumptions of normality (Shapiro–Wilk test, *p* > 0.05) and homogeneity of variance, so a main effects analysis of variance (ANOVA, *p* < 0.05) and Tukey’s HSD test were used to assess the differences between the groups. Each black circle represents the yield for a given sample. (ns: no significance; ****: *p* < 0.0001).

Figure 1 shows the yield results of obtaining RNA concerning the platforms and RNA isolation methods used. The NanoDrop Lite Spectrophotometer, Qubit 2.0 Fluorometer, and Agilent 2100 Bioanalyzer were compared for their advisability to provide a reasonable measurement of RNA concentration obtained from all samples; the three methods were able to detect RNA in all recently processed samples (Figure 1A). However, the NanoDrop platform showed the highest quantification values in all extraction methods (22.6 to 268.9 ng/µL), and the Qubit and the Bioanalyzer platforms resulted in statistically similar measurements (2.1 to 50.6 ng/µL). Regarding the coefficient of variation (CV), the Qubit 2.0 platform obtained the smallest variations (7.2% CV) compared to the Bioanalyzer (10.9% CV) and the NanoDrop (43.1% CV) platforms. Thus, the comparative analysis continued based on Qubit 2.0 quantification due to the offered sensitivity. In the comparative analysis of RNA isolation methods, RNA yields from TRIzol-treated samples were significantly higher than the other methods (59 ± 11 ng/100 µL or 10 mg V) (Figure 1B). No significant difference between the High Pure RNA Isolation kit (26 ± 9 ng/100 µL or 10 mg V) and the GeneJET RNA Purification kit (24 ± 12 ng/100 µL or 10 mg V) protocols was found. The Dynabeads mRNA DIRECT kit protocol provided the lowest yields (5 ± 4 µg/100 µL or 10 mg V) (Figure 1B). Subsequently, the TRIzol method was used for the subsequent analysis of the yield of obtaining RNA regarding the type of sample and the species.

**Figure 2:**
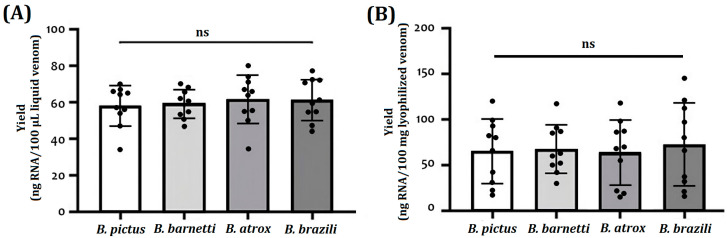
Comparison of RNA yield obtained from freshly collected (**A**) and lyophilized venom-derived (**B**) RNA isolated by TRIzol reagent protocol. The analysis of variance (ANOVA *p* < 0.05) and Tukey’s test (*p* < 0.05) were used to assess the differences between the species. Each black circle represents the yield for a given sample. (ns: no significance).

Table 1 and Figure 2 show the yields (ng/amount of biological sample) of total RNA extracted from TRIzol-treated venoms and venom glands. For fresh liquid venoms, the total RNA extracted yielded 47 to 75 ng of RNA per 100 µL of venom (Table 1, Figure 2A). For lyophilized venoms, samples yielded about 27 ± 119 ng of RNA per 10 mg of sample (Figure 2B). In such a way, taking into account the starting amounts of venom, a higher yield was achieved with the liquid samples. The RNA obtained from venom glands had the highest yield; the values range between 95 to 187 ng RNA/mg tissue. High intraspecific heterogeneity (CV: 15.7 to 78.0%) was observed in RNA yields.

**Figure 3:**
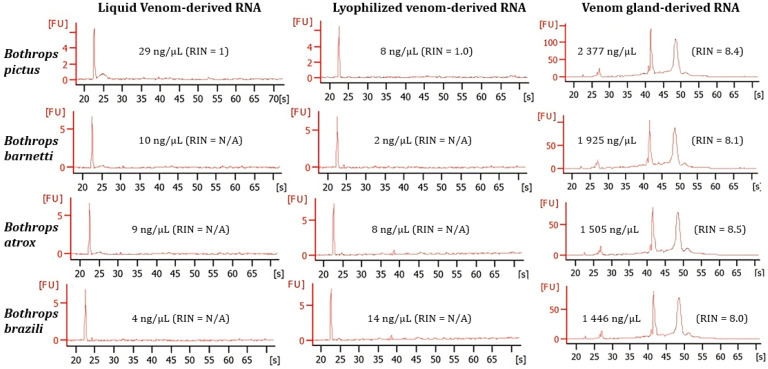
Electropherograms obtained with the Bioanalyzer 2100. Examples of the profile from fresh liquid venom samples, lyophilized venom samples and venom gland samples for each species. All electropherograms include the corresponding measuring of RNA concentration and the RNA integrity number (RIN).

Neither lyophilized venom RNA nor fresh liquid venom RNA had detectable RIN (RNA integrity number) values, while RIN values of venom gland RNA were above 8.0 (Figure 3). However, the lowest concentrations were recorded in both lyophilized and fresh venom on the Bioanalyzer (from 2 ng/µL to 29 ng/µL) compared to the venom gland (from 1446 ng/µL to 2377 ng/µL).

### 2.2. cDNA Synthesis

**Figure 4:**
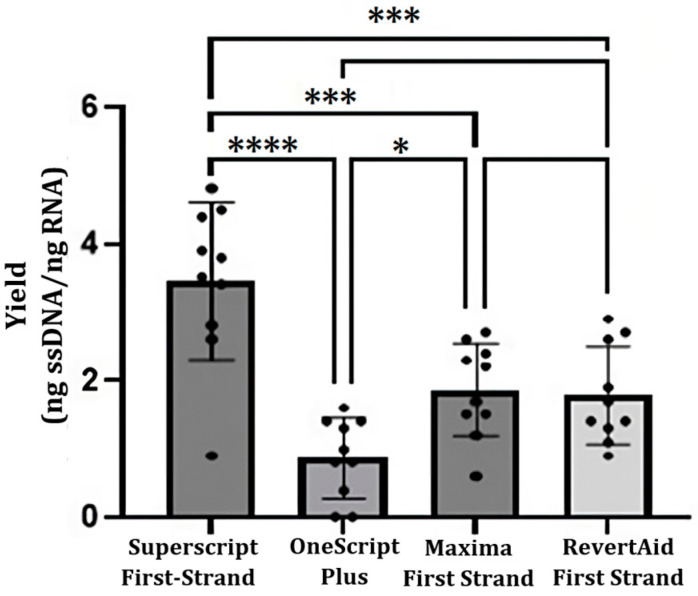
Comparison of cDNA yields using three different synthesis kits. The analysis of variance (ANOVA *p* < 0.05) and Tukey’s test (*p* < 0.05) were used to assess the differences between the commercial kits. Each black circle represents the yield for a given sample. (ns: no significance; *: *p* < 0.05; ***: *p* < 0.001; ****: *p* < 0.0001).

cDNA synthesis was possible using TRIzol method-RNA and the four cDNA synthesis kits, cDNA yields (ng cDNA per ng RNA) are shown in Figure 4. Notably, the SuperScript First-Strand Synthesis System kit produced high amounts of cDNA (3.2 ± 1.2). No significative differences were recorded between Maxima First Strand (1.9 ± 0.9) and RevertAid First Strand (1.6 ± 0.4) kits. OneScript Plus cDNA Synthesis Kit produced the least amount of cDNA (0.8 ± 0.7). Nevertheless, the combination of the four methods of RNA purification and the four cDNA synthesis kits was performed, and the results are shown in Table 2. High performance values were obtained with the SuperScript First-Strand Synthesis System kit in combination with the four RNA purification methods; the highest value was from a combination of the SuperScript First-Strand Synthesis System kit with Dynabeads mRNA DIRECT (4.8 ± 2.0).

### 2.3. PCR Amplification

**Figure 5:**
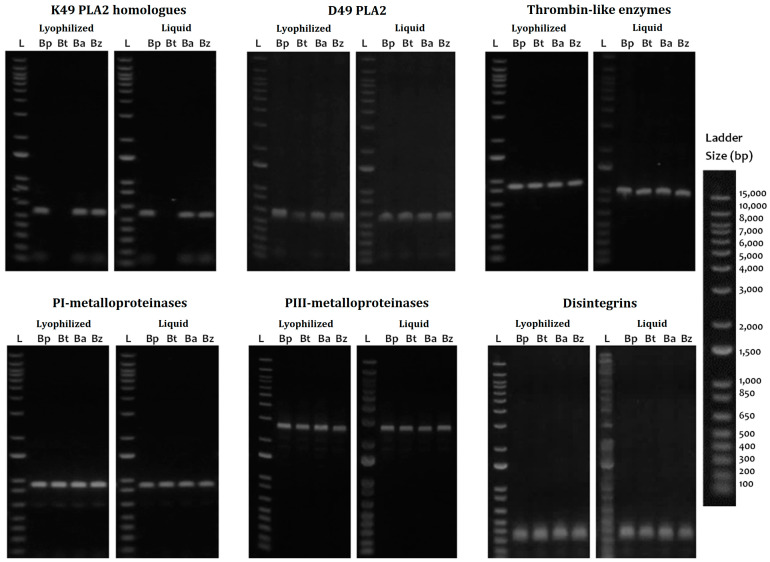
PCR amplification of transcripts from cDNA derived from freshly and lyophilized *Bothrops* venom samples. Qualitatively similar PCR products were amplified from cDNA from lyophilized and fresh venoms from *B. pictus* (Bp), *B. barnetti* (Bt), *B. atrox* (Ba), and *B. brazili* (Bz) snakes, using primers complementary to classical D49-PLA2, K49-PLA2 homolog, thrombin-like enzyme, P-I and P-III metalloproteinases, and hypothetical disintegrins transcripts. Ladder (L): 1 Kb Plus DNA Ladder (Cat# 10787018, Thermo Fisher, Waltham, MA, USA).

**Figure 6:**
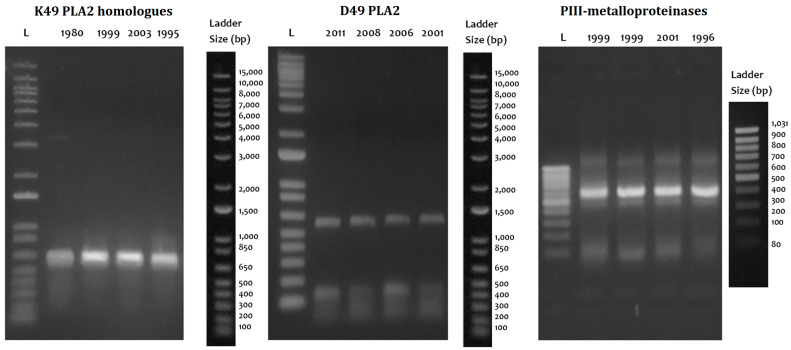
Stability of extracellular mRNA contained in long-term storage lyophilized venoms. Qualitatively similar PCR products were amplified from cDNA from lyophilized and fresh venoms stored for 5 to 30 years. Ladders: 1 Kb Plus DNA Ladder (Cat# 10787018, Thermo Fisher) and GeneRuler 100 bp DNA Ladder (Cat# SM0383, Thermo Fisher).

The cDNA utility was demonstrated with the amplification of the coding region for six of the major clinically relevant toxins such as thrombin-like enzymes (TLE), P-I metalloproteinases (P-I SVMP), P-III metalloproteinase fragments (P-III SVMP), classical D49 phospholipases A2 (D49-PLA2), K49-PLA2 homologs (K49-PLA2), and disintegrins (DIS) (Figure 5). TLEs, P-III SVMPs, PLA2, and DIS were completely amplified in all four species evaluated in both the lyophilized venom and the fresh liquid venom; however, the intensity of the bands was higher in the fresh liquid venom. K49-PLA2-amplified products could not be obtained from *B. barnetti* venom, and for this cDNA, variation in the intensity of the bands was appreciated; likewise, the P-I SVMP from the fresh liquid venom of *B. brazili* was not amplified. Figure 6 shows the results of long-term storage *B. atrox* venoms PCR amplification. For these samples, Qubit 2.0 Fluorometer could not detect any ribonucleic acid (<0.20 ng/µL) in most of the samples tested. Nonetheless, cDNA transcripts encoding D49-PLA2, K49-PLA2, and P-III SVMP (partially) were amplified.

### 2.4. Cloning and Sequences Identification

**Figure 7:**
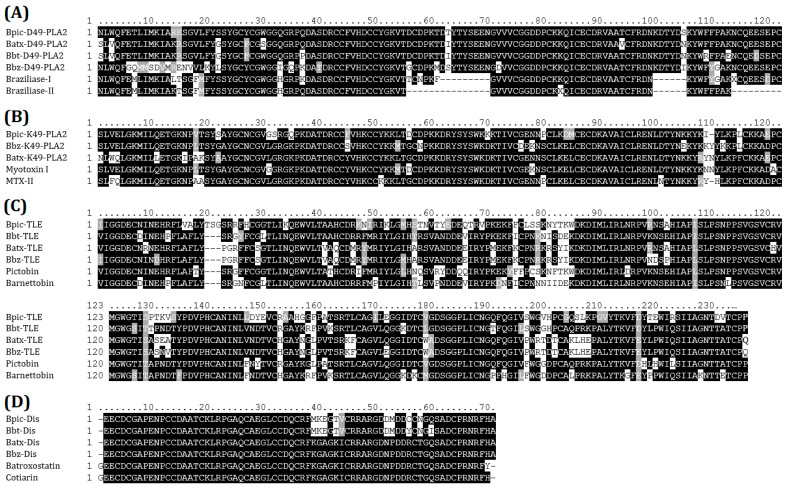
Amino acid sequence alignment of deduced amino acid sequences of D49-PLA2 (**A**), K49-PLA2 homologs (**B**), thrombin-like enzymes (**C**), and hypothetical disintegrins (**D**) from *Bothrops* species. UniProtKB accession numbers are as follows: Braziliase-I (P0DUN3) and Braziliase-II (P0DUN4) from *Bothrops brazili*, Myotoxin I (Q6JK69) from *B. atrox*, MTX-II (I6L8L6) from *B. brazili*, Pictobin (U5YCR8) from *B. pictus*, and Barnettobin (K4LLQ2) from *B*. *barnetti*, Batroxostatin (P18618) from *B. atrox*, Cotiarin (P31988) from *B. cotiara*. Bpic: *B. pictus*, Bbt: *B. barnetti*, Batx: *B. atrox*, Bbz: *B. brazili*.

**Figure 8:**
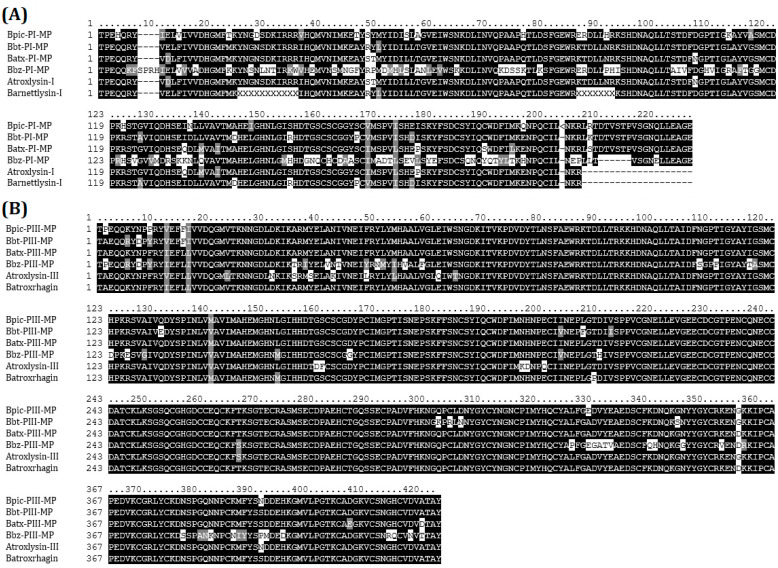
Amino acid sequence alignment of deduced amino acid sequences of P-I (**A**) and P-III (**B**) metalloproteinases from *Bothrops* species. UniProtKB accession numbers are as follows: Atroxlysin-1 (P85420) from *B. atrox*, Barnettlysin (P86976) from *B. barnetti*, Atroxlysin-III (A0A1S6K7T1) and Batroxrhagin (A0A0K2JNB8) from *B. atrox*. Bpic: *B. pictus*, Bbt: *B. barnetti*, Batx: *B. atrox*,Bbz: *B. brazili*.

PCR products obtained from fresh liquid venom were successfully cloned into the vector pCR2.1-TOPO in *Escherichia coli* cells TOP10′F. Culturing of cells transformed with TOPO-TA reactions resulted in an average efficiency of 4.2 × 10^6^ CFU/μg plasmid DNA. The identity of nucleotide sequences was confirmed by Sanger sequencing. We obtained one sequence for each amplified product corroborating the amplification of one isoform per each amplicon. The sequences obtained were then aligned and analyzed for homology with other sequences in the NCBI database. Multiple alignment from D49-PLA2s (Figure 7A), K49-PLA2s (Figure 7B), SVSPs (Figure 7C), DIS (Figure 7D), P-I SVMPs (Figure 8A), and P-I SVMPs (Figure 8B); all the sequences had high similarity with those of the database. The main regions of each protein involved in toxicity were conserved. Protein homologs clones were sequenced, being identical to the sequence directly obtained from the transcripts.

## 3. Discussion

A complementary approach to the biochemical and biological characterization of toxins isolated from snake venoms is the characterization at the transcriptional level (mRNA). For this purpose, it is important to have efficient methods for mRNA or total RNA isolation. Several studies report the efficient isolation of RNA by different methods from snake venom, highlighting its suitability for the identification of protein-coding transcripts [13,21,28]. The success of the isolation method depends on the quantity and integrity of RNA obtained. Few studies have evaluated snake venom- or venom-gland-derived RNA yield and quality [10,13,21]. Although studies are reporting on the molecular characterization of *Bothrops* venom proteins from total extracellular RNA [20,25,29,30], none report yields of RNA obtained.

To our knowledge, there is only one study comparing different commercial kits of snake venom RNA isolation from fresh and lyophilized snake venoms [13]. Like our results (0.50–6.1 µg of RNA), these authors obtained better yield using the TRIzol method for RNA purification (1.10–13.60 µg of RNA) from *Crotalus* and *Sistrurus* venom. The method based on GITC (guanidine thiocyanate), as TRIzol reagent protocol, offered better results for both sample types. Similar results were obtained in *Crotalus* venoms, where the TRIzol protocol was widely superior to the other five methods, including those that are based on silica columns, glass microfibers, and magnetic beads, although it was not accompanied by statistical support [13]. Other studies comparing RNA methods from different types of biological samples (viruses, fungi, plant tissues, and body fluids, among others) also concluded that the TRIzol-based method offered higher yields [31,32,33,34].

Despite the higher performance of the TRIzol reagent protocol, physicochemical properties of adipose tissue, protein-rich source (as in the snake venom), and phenol can impair the RNA purity obtained. As expected, the High Pure RNA Isolation and GeneJET RNA Purification kits provided readings (A260/280 ratio: ~1.85) significantly higher than the TRIzol reagent protocol (A260/280 ratio: ~1.72). Some studies have applied diverse strategies to overcome the relatively low purity, such as optimizations in volume used, the use of a column-based secondary purification, or additional wash steps [35,36,37]. Considering that venom is not an RNA-rich source, we must decide if these protocol modifications (at the expense of reduced yield) are required for the post-extraction techniques.

No statistically significant differences in RNA yield of lyophilized and fresh samples were observed between species (Figure 1A,B); however, high coefficients of variation (up to 40%) have revealed a high intraspecific heterogeneity. This variability could be associated with differential gene expression, which is strongly linked to various biological factors such as ontogeny, sexual dimorphism, and ecological distribution [38]. Variations in global gene expression are associated with the venom production cycle. After venom release, the venom gland manifests a rapid and extensive sequential upregulation of protein transcription, translation, processing, and secretion [39].

Quantitation based on UV light absorption (as in the NanoDrop) can overestimate the RNA concentration due to certain interfering molecules (which have a similar absorption spectrum), such as phenolic compounds (present in the TRIzol reagent), DNA, and proteins (abundant in the venom) [40,41]. Fluorescence-based quantification uses dyes that specifically bind to one type of nucleic acid (single- or double-stranded DNA or RNA); it also includes the use of standard samples (of known concentration). Therefore, the fluorometric readings obtained in the present study provide a greater closeness to the actual concentrations of the isolated RNA.

Additionally, the high quality of RNA is a prerequisite for the viability of various molecular biology techniques, such as cDNA library construction, real-time PCR, gene cloning, targeted mRNA- and high-throughput sequencing, northern hybridization analysis, etc. Although the three platforms used can measure RNA, only the Bioanalyzer can reliably report the RNA quality (degraded or intact) through an RNA Integrity Number (RIN). We assessed the RNA quality from lyophilized venom, liquid venom, and venom gland samples. The disparity between RIN values suggests that the venom samples provide considerably degraded RNA (Figure 3). The RIN value is inferred based on the ratio of ribosomal RNAs (18 S and 28 S in eukaryotic samples). Gland cells can secrete nucleic acids through the extracellular vesicles [42,43]. We hypothesize that due to the large size of ribosomal RNAs, their secretion into the glandular lumen is limited. Therefore, in these cases, the RIN value may not be a direct indicator of the quality of the venom RNA.

We synthesized 8 to 35 ng of cDNA per 10 ng of RNA using different cDNA synthesis kits. Currier et al. (2012) reported yields close to 12 μg of cDNA from 10 ng of mRNA. This is due to the lower proportion of mRNA versus ribosomal RNA and transfer RNA. However, in any combination used of the extraction method and cDNA synthesis kit, the amounts of cDNA obtained were sufficient for the PCR amplification. Likewise, it was fascinating to be able to synthesize cDNA from venom samples that were lyophilized several years ago. The unexpected stability of the lyophilized venom-derived mRNA has been reported by other authors [10,13,22].

Cataloging of toxins can be carried out at the transcript level (characterization molecular). Omics approaches revealed that *Bothrops* snake venoms are composed predominantly of phospholipase A2, metalloproteinases, serine proteinases, disintegrins, L-amino acid oxidases, C-type lectin-like proteins, and hyaluronidases [23,44,45,46,47]. We amplified cDNA transcripts encoding K49-PLA2s, D49-PLA2s, TLEs, P-I and P-III SVMPs, and DIS. Other works have also amplified cDNA from venoms of the snakes included in this study, which has allowed the molecular characterization of the BaMtx [14], Barnettobin [27], Pictobin [29], Atroxlysin-III [25], Hyal-Ba [30], Bpic-LAAO [20], Pictolysin-III [17], and BaPer-PLA2 [26].

## 4. Materials and Methods

### 4.1. Venoms and Venom Gland Collection

Venom samples were obtained from nineteen adult pit-viper snakes of undetermined sex, *Bothrops pictus* (“jergón de costa”), *Bothrops barnetti* (“sancarranca”), *Bothrops brazili* (“jergón-shushupe”), and *Bothrops atrox* (“jergón de selva”) snakes. Five snakes of each species were used for crude venom collection. All snakes were maintained in individual terrariums with temperature- and humidity-monitored environments at the Oswaldo Meneses Serpentarium of the Natural History Museum (Universidad Nacional Mayor de San Marcos, Lima-Peru). Venom samples were collected twice from each specimen with an interval of two months between each milking event. Venom milking was performed three days after the feeding date. The venoms were placed in microtubes containing TRIzol or RNAlater, depending on the protocol to be followed. In addition, long-term storage (from 1980 to 2011) of lyophilized venoms (about 30 samples per species) was provided by the Laboratory of Molecular Biology from the Biological Sciences Faculty (UNMSM).

From 2017 to 2022, venom glands were collected from nine snakes (three *B. pictus*, three *B. atrox*, two *B. brazili*, and one *B. barnetti*) according to the protocol recommended by [23] and dissected into small pieces. These specimens were fatally injured by farmers and reported to the serpentarium for identification. The excised gland tissues were mixed briefly with RNAlater solution at 4 °C and stored at −80 °C until use. All procedures were performed according to local protocols for breeding venomous snakes and the guidelines of the “WHO Guideline for the Production Control and Regulation of Snake Antivenom Immunoglobulins” [48]. The handling of live snakes for venom extraction has been approved by the ethics committee of FCB-UNMSM (Code N°: 014-2022-CBE-FCB-UNMSM).

### 4.2. RNA Isolation

RNA was isolated from fresh liquid (100 µL) samples and previously stored lyophilized (10 mg) venom samples, as well as from fresh venom glands, using four commercially available kits according to the manufacturer’s instructions. Specifically, TRIzol reagent (Cat# 15596026, Invitrogen, CA, USA) was used to isolate total RNA from venom and gland venoms (only protocol applied to glands) with slight modifications [13]. For comparison, glass fiber membrane-based High Pure RNA Isolation kit (Cat# 11828665001, Roche Applied Science, Mannheim, Germany) and silica column-based GeneJET RNA Purification kit (Thermo Fisher Scientific Inc, Lithuania, MA, USA) were used to isolate total RNA from RNAlater-treated venom samples, where magnetic bead-based Dynabeads^®^ mRNA DIRECT™ kit (Cat# 61011, Invitrogen, Grand Island, NY, USA) was used to isolate poly(A) RNA. All RNA extractions were performed in triplicate, and samples were immediately used in cDNA synthesis.

### 4.3. cDNA Synthesis

All purified RNA samples were processed into single-stranded cDNA using oligo(dT) primers and different kits according to the manufacturer’s instructions: OneScript™ Plus cDNA Synthesis kit (Applied Biological Materials Inc, Richmond, BC, Canada), Maxima First Strand cDNA Synthesis kit (Thermo Fisher Scientific Inc, Lithuania, MA, USA), RevertAid First Strand cDNA Synthesis kit (Thermo Fisher Scientific Inc, Lithuania, MA, USA), and SuperScript™ First-Strand Synthesis System (Invitrogen, Grand Island, NY, USA).

### 4.4. Nucleic Acids Quality Control

RNA and DNA concentrations were measured in the NanoDrop Lite Spectrophotometer (Thermo Fisher Scientific), Qubit 2.0 Fluorometer (Invitrogen, Carlsbad, CA, USA), and Agilent 2100 Bioanalyzer (Agilent Technologies, Foster City, CA) according to manufacturer specifications. RNA purity was roughly estimated by measuring the absorbance ratios (260/280 and 260/230) using the NanoDrop Spectrophotometer. The RNA profile and integrity of all TRIzol-treated samples from venoms and venom glands were assessed using the Agilent 2100 Bioanalyzer.

### 4.5. Endpoint PCR Amplification

Primers were manually designed to amplify mRNA encoding *Bothrops* thrombin-like enzymes, P-III and P-I metalloproteinases, disintegrins, phospholipases A2, and PLA2 homologs (Table S1). Synthesized cDNA was amplified by PCR using the Invitrogen™ Platinum™ II Taq Hot-Start DNA Polymerase. PCR reactions were prepared in 20 μL volumes containing 10 ng cDNA, 0.5 μM of each primer, 0.5 μM dNTP mix, Platinum II PCR buffer, and 0.8 units Platinum II HS DNA polymerase. Amplification was performed as follows: initial denaturation step at 94 °C for 2 min, thirty cycles of 94 °C for 15 s, 60 °C for 15 s, and 68 °C for 15 s/kb. The amplified products were visualized on a 1% TAE/agarose gel with GelRed Stain 100X.

### 4.6. Cloning of Amplified Products and Sequencing

Adding 3′ A-overhangs was performed using the Platinum™ II Taq Hot-Start DNA Polymerase. cDNA amplified by Phusion™ DNA Polymerase according to manufacturer specifications. PCR products were inserted with the pCR2.1-TOPO vector following the TOPO-TA cloning strategy and transformed into One Shot™ *Escherichia coli* TOP10 competent cells, according to the manufacturer’s recommended protocol. Transformed *E. coli* cells were grown on Luria-Bertani (LB) agar plates overnight at 37 °C spiked with ampicillin (50 µg/mL) for selection. Positive colonies (n = 5) were analyzed by colony PCR amplification using M13 primers and the Platinum™ SuperFi II Green PCR Master Mix. Only 1 positive band (resulting in the estimated size) was excised and purified, using the GeneJET Gel Extraction kit, from the plate tested. Purified amplicons were sequenced by Macrogen, Inc. (Seoul, South Korea) on an ABI 3730 XL automated sequencer. All inserts were amplified and sequenced using M13 primers from 100 pg plasmid DNA.

### 4.7. Sequence Analysis

cDNA and sequences were compared with other nucleotide sequences deposited in GenBank (https://www.ncbi.nlm.nih.gov/genbank/, accessed on 15 May 2023). Protein sequences were deduced using the Translate tool (http://web.expasy.org/translate/, accessed on 30 May 2023), and UniProtKB http://www.uniprot.org/, accessed on 30 May 2023) was used for comparison. The multiple sequence alignments (MSA) for all protein families were performed by MUSCLE algorithm [49] within the MEGA-X (v11.0.13) program using the program default parameters [50]. Some alignments were manually modified to adjust with the numbering system proposed by different works.

### 4.8. Statistical Data Analyses

The data was collected in MS Excel and statistically analyzed using GraphPad Prism software v9.5.1. Data is expressed as the mean and standard deviation. For each sample type, significant differences in the Qubit readings of RNA were evaluated via one-way analysis of variance (ANOVA) followed by Tukey’s (post-hoc) multiple comparisons tests. In cases where the RNA concentrations did not meet the assumptions of normality (Shapiro–Wilk test) and homogeneity of variance, so to assess the differences between the groups, non-parametric Kruskal–Wallis test and multiple comparisons with Dunn’s test were used. For all analytical proposes, statistical significance was declared at *p* < 0.05.

## 5. Conclusions

In conclusion, we confirmed that the stability, purity, and quantity of the Peruvian *Bothrops* venom-derived mRNA are enough to consider the venom as an alternative source to partially explore the glandular transcriptome. This avoids the need to sacrifice the animals, especially with species in a vulnerable state such as *Bothrops pictus* and *Bothrops barnetti*. On the other hand, the long-lasting stability of RNA in lyophilized venom is surprising, which may even reduce the need for venom collection events. Finally, we hope that these results will facilitate work based on snake venom RNA to determine the composition of a little-studied snake through the identification and molecular characterization of unknown/known toxins.

## Figures and Tables

**Table 1:** RNA yields from *Bothrops* snake venoms and gland venoms.

RNA Isolation Protocols	RNA Yield * per Species			
	*B. pictus*	*B. barnetti*	*B. atrox*	*B. brazili*
Freshly collected venoms (ng RNA/100 µL venom)	58 ± 11	59 ± 8	62 ± 13	61 ± 11
Lyophilized venoms RNA Yield (ng RNA/10 mg venom)	65 ± 35	68 ± 27	83 ± 21	73 ± 46
Snake venom glands (ng RNA/mg tissue)	120 ± 25	143 ± 26	156 ± 31	136 ± 28

* Values were determined using a Qubit 2.0 Fluorometer. The data are shown as mean ± SD. Each sample was processed in triplicate.

**Table 2:** Venom-derived cDNA yields obtained by different cDNA synthesis kits. The data are shown as mean ± SD.

	cDNA Yield (ng cDNA ^1^/ng RNA ^1^)			
Isolation Method	TRIzol Reagent Protocol	High Pure RNA Isolation Kit	GeneJET RNA Purification Kit	Dynabeads mRNA DIRECT Kit
SuperScript First-Strand Synthesis System	3.2 ± 1.2	2.1 ± 1.5	2.7 ± 1.1	4.8 ± 2.0
OneScript Plus cDNA Synthesis Kit	0.8 ± 0.7	0.6 ± 0.4	0.9 ± 0.3	2.0 ± 1.0
Maxima First Strand cDNA Synthesis Kit	1.9 ± 0.9	2.1 ± 0.6	n.d.	n.d.
RevertAid First Strand cDNA Synthesis Kit	1.6 ± 0.4	2.4 ± 0.2	n.d.	n.d.

^1^ RNA and cDNA amounts were determined by fluorometry. Biological for RNA isolation. Each sample was treated for at least per triplicate. n.d.: not determined.

## Data Availability

Not applicable.

## Supplementary Materials

The following supporting information can be downloaded at https://www.mdpi.com/article/10.3390/ijms241311161/s1.
