# Supplementary material for: Comparison of Four Methods of RNA Extraction and cDNA Synthesis from The Venom of Peruvian Snakes of the Genus Bothrops of Clinical Importance
Source: Int J Mol Sci. 2023 Jul 6;24(13):11161. doi: 10.3390/ijms241311161 (PMC10342303; doi:10.3390/ijms241311161)
Supplement: Supplementary file 1 [file ijms-24-11161-s001.zip › ijms-2460588-supplementary.pdf]

SUPPLEMENTARY INFORMATION

Table S1. List of primers used for amplification of six toxin-encoding transcripts

Specificity	5′ → 3′ Forward primer sequence	5′ → 3′ Reverse primer sequence
SVSP	TCTTCTGAACTGGTCATTGG or AGTCATTGGAGGTGATGAATG	TTTCCTGCAATAATGCTCTG or CGGGGGGCAAGTCTCAGTTGT
PIII-SVMP	TCCAGCAAATCCAGCCTCC	AGGTGTAGCCACATCAAC
PI-SVMP	AGCTCTATAATCCTGGAATCTG	ACAGTATCTGTTCTCAAGCG
Asp49 PLA_2	TGGACCACGTTAAACTCTGCAG	GGAGTCAGACCATGCTAA
Lys49 PLA_2 homologue	AACCTGTGGCAATTGGGGAAG	TTAGCATGGCTCTGCCTTCT
Disintegrin	GAAGAATGTGACTGTGGC	TCTGGGACAGTCAGCAG

Uncropped gels

Relative to Figure 5

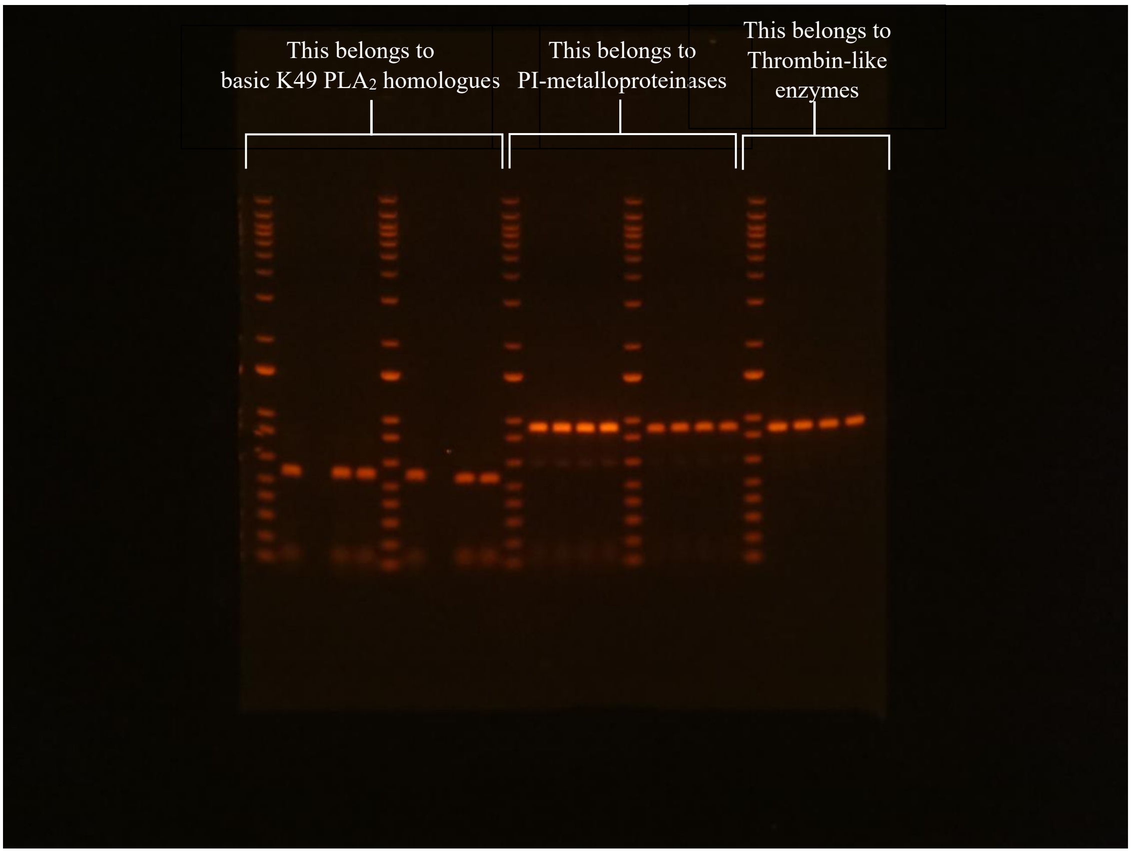

Relative to Figure 5

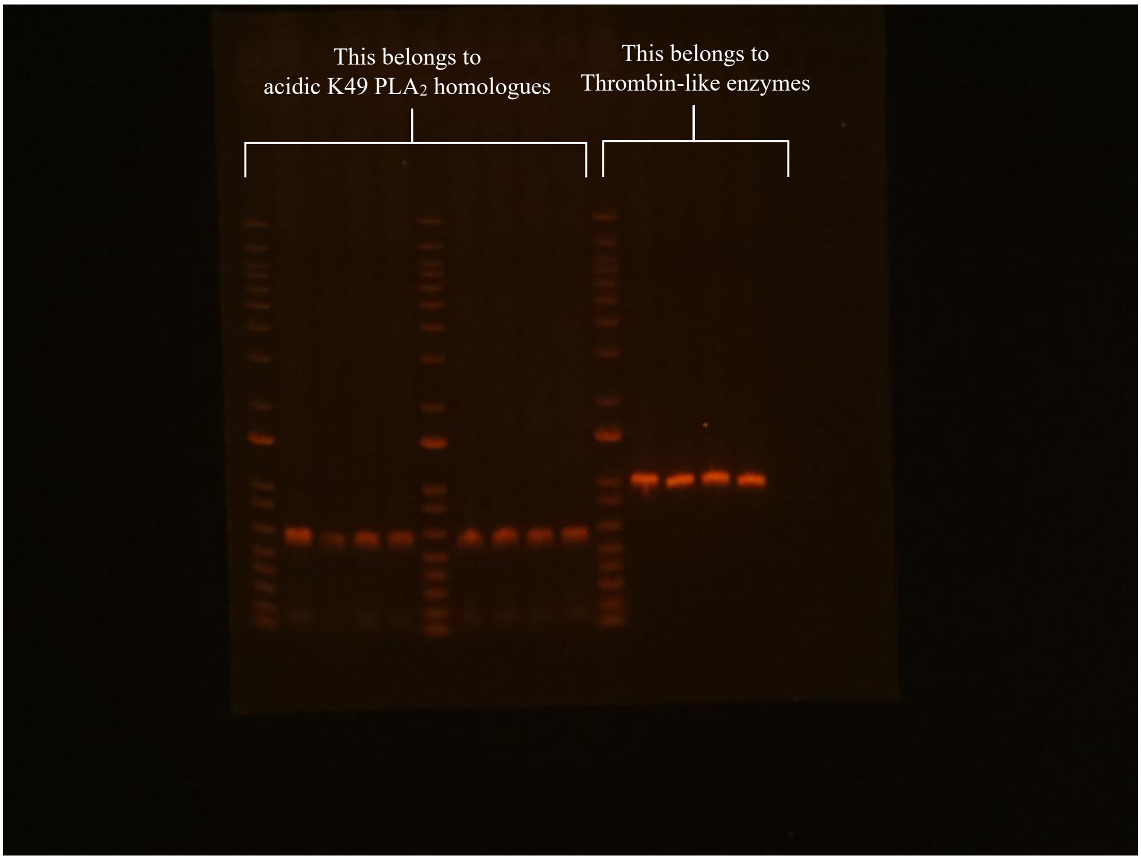

Relative to Figure 5

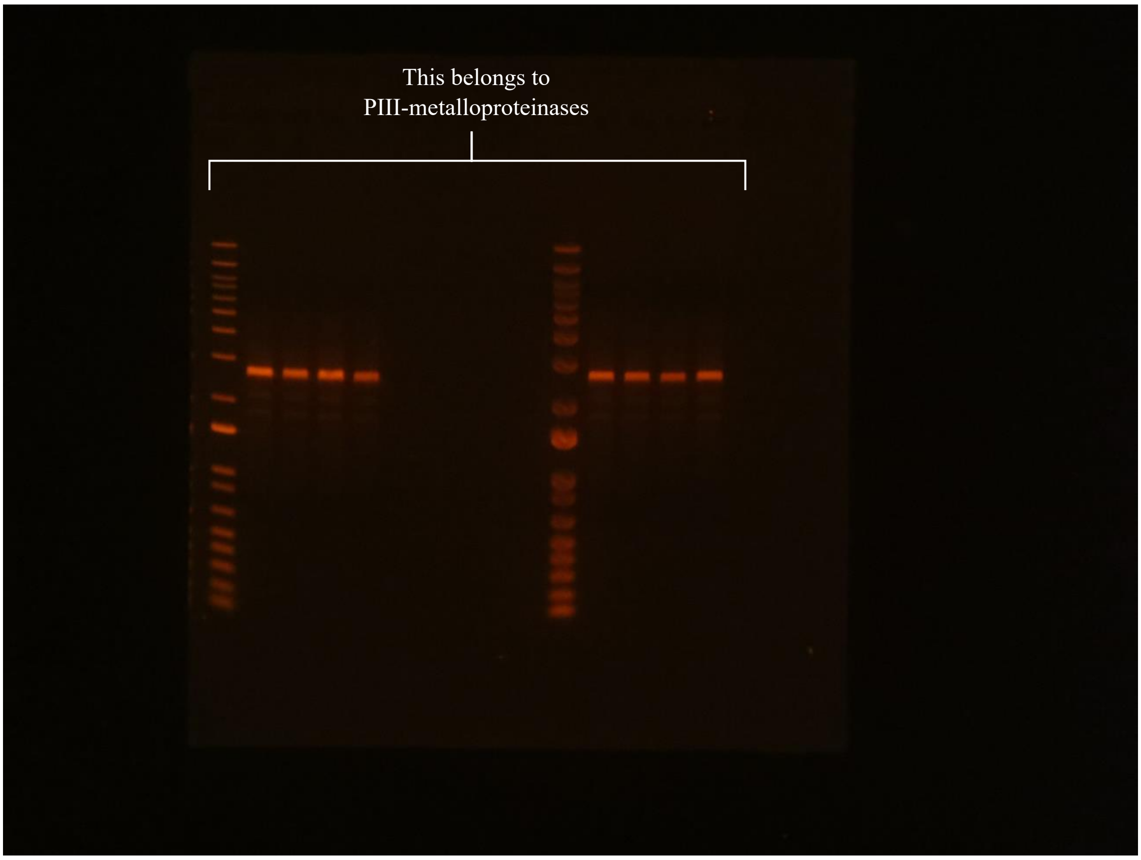

Relative to Figure 5

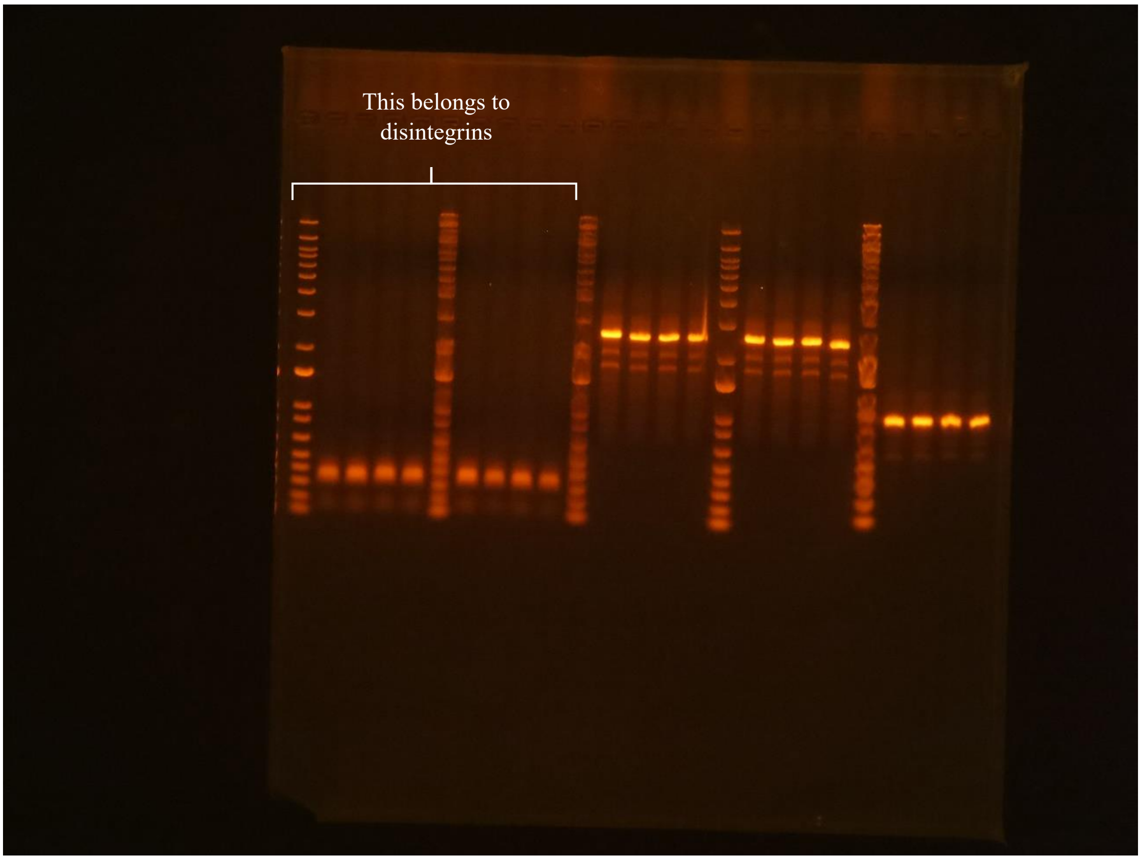

Relative to Figure 6

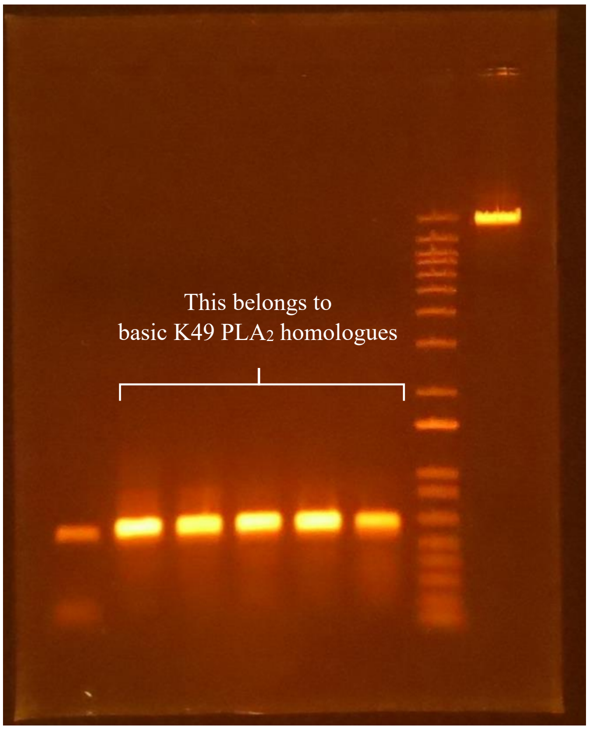

Relative to Figure 6

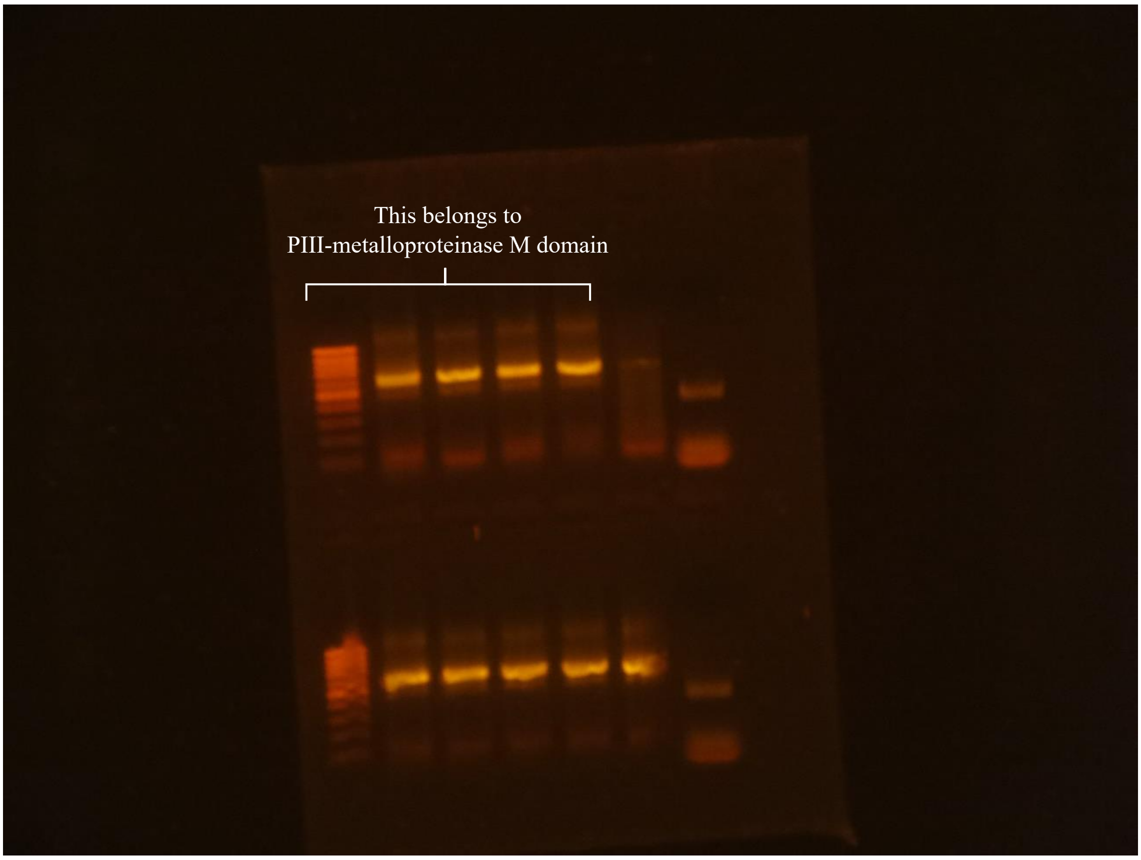

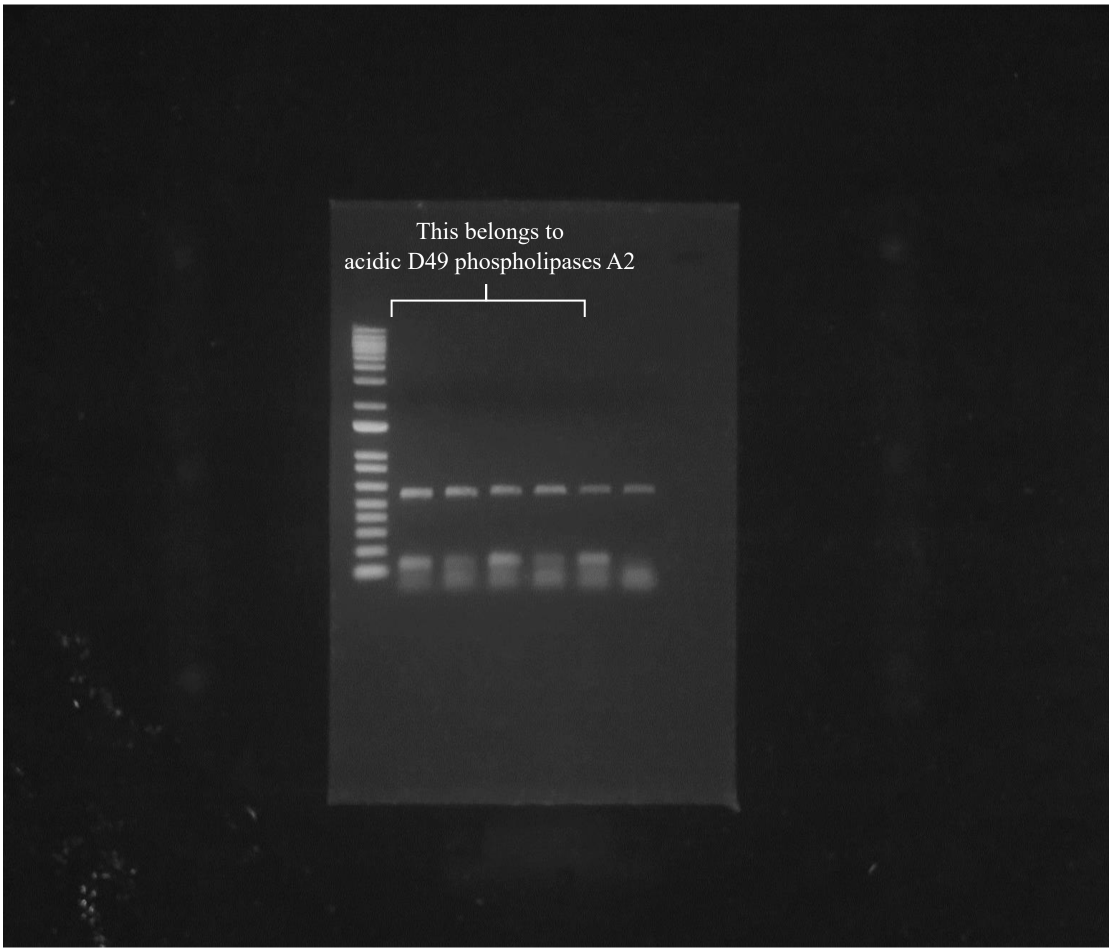
This belongs to
acidic D49 phospholipases A2
